# For the rural curious: mixed methods evaluation of a rural pharmacy practice elective

**DOI:** 10.1186/s12909-024-05539-3

**Published:** 2024-05-24

**Authors:** Timothy P. Stratton

**Affiliations:** University of Minnesota College of Pharmacy, Duluth, MN USA

**Keywords:** Rural pharmacy, Pharmacists, Rural health, Pharmacy education

## Abstract

**Background:**

As of 2020, 20% of people residing in the United States of America (U.S.) lived in rural communities. Despite rural residents tending to be older, poorer, and having greater disease burden than their urban counterparts, the number of rural primary care providers continues to decline. Nearly 66% of U.S. Primary Care Health Professional Shortage Areas are designated as rural. Pharmacists can help address this shortage of rural primary care providers, often serving as providers of first-contact care; however, only 12% of U.S. pharmacists practice in rural communities. To help address this gap, in 2022 an elective Rural Pharmacy course was created at the University of Minnesota College of Pharmacy by a faculty member who has rural practice experience.

**Methods:**

The course combines formal lectures, guest presentations by rural pharmacists and student interviews with additional rural pharmacists. For the 42 students enrolled in the course in 2022 and 2023, non-parametric statistics were used to compare the percentage of students who were raised in rural communities or who otherwise had extensive exposure to rural, and compare student interest ratings (1 to 7) about practicing/living rural at the beginning and end of the course. Students also wrote end-of-course reflection papers, commenting on the course and their interviews with rural pharmacists.

**Results:**

Across both years, 45% of the enrolled students had previous experience in rural communities. The net change in Rural Interest scores among students completing both questionnaires was + 5 in 2022 and + 2 in 2023, both non-significant differences. The largest shifts in student interest were from “Not Sure” at the start of the course to “Interested” or “Not Interested” at the end of the course, and from “Interested” to “Very Interested.” In their reflection papers nearly 60% of students reported being most impressed by their interviews with rural pharmacists.

**Conclusions:**

A course addressing the benefits and challenges of practicing pharmacy in rural communities was well-received by pharmacy students. Even students who have little interest in living in a rural community can benefit from being introduced to rural culture, enabling them to provide more culturally-responsive care for patients from rural communities.

## Introduction

The Unites States (U.S.) Census Bureau redefined “rural” for the 2020 census as communities with populations of fewer than 5,000 people (fewer than 2,000 housing units) and located more than 1.5 miles (2.4 km) from a high-density urban area. Based on this revised definition, 20% of the U.S. population in 2020 lived in rural communities [1]. The percentage of rural residents varied greatly by region, with only 11% of people in the West Region residing in rural areas, followed by 16% in the Northeast Region, 24% in the South Region and 26% in the Midwest Region [2]. At the extremes, fewer than 6% of people in California lived in rural areas, while nearly 65% of Vermont residents lived in rural areas [1]. In contrast, as of 2021 the U.S. Bureau of Labor Statistics reported that only 12% of the nation’s pharmacists practiced in nonmetro (rural) communities [3].

On average, rural residents tend to be older [4], poorer [5], experience greater disease burden [6] and lack health insurance or be underinsured [7] than residents of urban communities. The average age and disease burden among rural residents is increasing due to outmigration of young adults from rural to urban communities and the in-migration of older adults to rural communities following retirement [4]. Yet as the proportion of older residents in rural communities continues to increase, the availability of primary care providers in rural communities continues to decrease. Nearly 66% of Primary Care Health Professional Shortage Areas in 2023 are designated as rural [8]. Pharmacists can be part of the solution to address the existing and anticipated shortage of primary care providers in rural communities [9]. Rural pharmacists often serve as “providers of first-contact care” for patients who are seeking to self-treat a health condition [10]. Where self-treatment is inappropriate, the pharmacist is in a position to refer the patient to appropriate professional care.

This paper describes a new course taught in the Doctor of Pharmacy (PharmD) program in the University of Minnesota College of Pharmacy that introduces students to the unique benefits and challenges of practicing pharmacy in rural communities.

### One college, two campuses

The University of Minnesota (UMN) is a public, research-intensive (Carnegie R1) institution. The UMN College of Pharmacy opened on the Minneapolis campus in 1892 [11]. Prior to 2003, the College of Pharmacy included four departments: Experimental & Clinical Pharmacology, Medicinal Chemistry, Pharmaceutics, and Pharmacy Care & Health Systems. However, to address a shortage of pharmacists in Greater Minnesota – counties outside of the seven-county Minneapolis-St. Paul Twin Cities Metro Area [12] – in 2003 the College of Pharmacy expanded its program 150 miles (241 km) north to Duluth on the University of Minnesota Duluth campus, adding a fifth department to the College, Pharmacy Practice and Pharmaceutical Sciences (PPPS).

The specific multi-campus model used by the UMN College of Pharmacy is somewhat unique among multi-campus pharmacy programs in the U.S. The PPPS department includes faculty representing Biochemistry and each of the major Pharmacy disciplines (Clinical Pharmacy, Medicinal Chemistry, Pharmaceutics, Pharmacology, Pharmacy Practice, and Social & Administrative Pharmacy). Didactic courses within the College are taught using videoconferencing technology, with classroom presentations/lectures originating from either Minneapolis or Duluth and being broadcast to the other campus.

The mission of the PPPS department includes preparing pharmacists to provide patient care in rural and Indigenous communities [13]. PPPS faculty embody this mission in all four areas of an academic health professions program, highlighting the unique health needs of rural residents in their teaching, addressing these needs through community-based participatory research [14], conducting service activities in rural communities, and providing clinical services. Until 2022, however, no single course in the College of Pharmacy’s curriculum was devoted specifically to rural health.

### Rural pharmacy elective-course description and structure

To help address this gap in the College of Pharmacy curriculum, the author – a pharmacist who has practiced hospital, community and long-term care pharmacy in frontier/Indigenous communities in Alaska [10], Eastern Montana and Minnesota – created a two-credit elective course (two hours per week for 15 weeks) in Rural Pharmacy to introduce students to the benefits and challenges of living and practicing in rural communities. Development of the course was guided by the author’s teaching philosophy; to paraphrase Confucian philosopher Xun Kuang [15]: “Tell me and I will forget. Show me and I will remember. Involve me and I will understand.”

The Rural Pharmacy course was designed as a HyFlex course [16] that allows the learner to choose by which content delivery method they would like to learn. Learners in a Hyflex course may elect to attend a live class session in person in a classroom, may attend a live class session remotely via videoconference, or may learn online anytime. Each live class session is recorded to accommodate students who prefer to learn online during a given week, or throughout the entire course.

The Rural Pharmacy elective is a “modified” HyFlex design in that no in-person option is available. University of Minnesota College of Pharmacy faculty and students are accustomed to videoconferencing as a course delivery method, the college having used videoconference technology since 2003 to conduct live, in-person sessions for learners on campuses located 2.5 h apart from one other. Required and elective didactic courses delivered by videoconference are always recorded, enabling learners to view the recording at a more convenient time if they are unable to attend the live class session. Another reason that an in-person option for the Rural Pharmacy elective is not offered is that live course sessions are conducted in the evening to accommodate students from different years in the pharmacy program (P2 and P3) whose other courses are all on different schedules, and Minnesota’s frequent snowy and icy winter conditions are not always conducive to safe travel to and from campus, especially at night.

At the time of this writing, during the first three pre-clinical years University of Minnesota College of Pharmacy students are required to complete 15 credits of elective courses above and beyond their required courses. The Rural Pharmacy elective is open to students in the final two pre-clinical years of the PharmD program (P2 and P3), but enrollment is capped by the instructor at 25 students per offering. Live class sessions are conducted once weekly for two hours in the early evening by videoconference for all students, whether based in Duluth or in the Minneapolis-St. Paul Twin Cities area. The early evening hours avoid conflicts with students’ other courses, which are on different schedules between 8:00 am and 5:30 pm for both of the two years. Students are encouraged to attend as many live videoconference sessions as possible, especially when a guest presenter is scheduled; however, as noted above all class sessions are recorded for viewing or reviewing at a more convenient time. The recordings accommodate students who may be working in a pharmacy as a Pharmacy Intern or Pharmacy Technician at the time class is scheduled, or students who desire to review one or more recorded class sessions prior to the written midterm examination.

A University of Minnesota Post-Graduate Year 1 (PGY1) Rural Pharmacy Resident [17] serves as the Teaching Assistant for the course each year, participating in the live class sessions via videoconference. The Pharmacy Resident is based out of a rural community in central Minnesota, traveling to two other rural communities and providing comprehensive medication management services [18] to residents of all three communities. While maintaining patient confidentiality, the Resident shares with students their experiences caring for patients in rural communities, some stories being only a few hours old. In addition to regularly participating in live class sessions, the TA prepares and leads a class session on their own, and conducts the live session interviews with guest rural pharmacists as described below.

About half of the class sessions feature guest pharmacists who currently practice in rural communities, guests joining the live class sessions via videoconference. When a guest pharmacist is invited to participate in the course, the instructor provides the pharmacist with a list of potential interview questions that they would be asked to address during the class session. On rare occasions the visits with pharmacist(s) are pre-recorded either to better accommodate the pharmacist’s work schedule or because of time zone differences between Minnesota and the states where the pharmacists live/work. Pre-recorded interviews are played during the live class session, and students submit questions they would have asked the pharmacist had the pharmacist been able to join the class session in real time. Those questions are then summarized by the instructor and forwarded to the guest pharmacist to respond to as the pharmacist’s time allows. Pharmacists living and practicing in rural and Indigenous communities from throughout Minnesota and from as far away as Alaska have participated in the live sessions, either pre-recorded or in real time. In addition to rural pharmacists, guest presenters have included Advance Practice Nurses [19] from rural communities, and a Biologist who works with an Indigenous community on the impacts of climate change on the health of the community.

A variety of assessments are utilized in the course including reflection papers, an online multiple-choice/true–false/short answer midterm exam, written participation in online discussions, in-class student presentations and written summaries of interviews with pharmacists practicing in rural communities. The course is graded on a A,B,C,D,F letter grade scale. A total of 300 points are available across nine activities in the course, ranging in value from 5–50 points. The grading scale used in the course is the professional scale used in all of the college’s courses, an A grade being attained by students who earn at least 93% of the available points while students earning fewer than 60% of available points do not receive a passing grade. The possible number of points available on individual assignments are assigned by the instructor based on the amount of time and effort students are expected to expend on the assignment as well as the quality of each assignment’s deliverable.

At the start of the course students complete a brief 7-point Likert-type questionnaire regarding their familiarity with rural communities and interest in possibly practicing in a rural community. The questionnaires are confidential rather than anonymous as students complete the same questionnaire again at the end of the course. The course director uses student names to match start-of-course and end-of-course questionnaires to measure changes in student attitudes. Students also write a brief paper describing their experiences with rural communities and the reason for their interest in learning (or learning more) about living and practicing in rural communities. The instructor uses this information to tailor presentations in the course for the entire class based on the students’ familiarity with rural communities. This information also familiarizes the instructor with students’ backgrounds, enabling the instructor to invite specific students to share their rural experiences as relevant opportunities arise during live class sessions. The initial questionnaire and interest paper collectively constitute 8.37% of the course grade.

The online midterm examination is based on material provided in the textbook [20] or during instructor or Resident presentations. Students are tested on their knowledge about what constitutes “rural” as defined by several different U.S. government agencies, rural culture, challenges in rural public health, and opportunities and challenges related to practicing pharmacy in rural communities. The midterm exam score constitutes 16.7% of the course grade.

As mentioned previously, the HyFlex nature of the course accommodates students who are unable to attend the live videoconference sessions. All students, however, participate in weekly written online discussions based on the live videoconference session from that week. Live sessions are recorded so that any student may view and listen to the session at their leisure. In the online discussion, students are asked to respond to an instructor-generated question based on that week’s live class session. Students are asked to post their response first, then comment on the response of at least one other classmate. The Canvas learning management system [21] facilitates this learning approach, providing the instructor the option to require a student to post their response before reading the responses of classmates. Students who post their responses by the weekly deadline receive full participation credit for the week, rather than being graded on the length of their response or on the number of responses they make to classmates’ postings. As a HyFlex course, students are not awarded extra points for attending the live videoconference session, nor are they penalized for not participating in the live videoconference session. Participation constitutes 16.7% of the course grade.

Indigenous people began living in what today is referred to as Minnesota some 13,000 years ago. Among the earliest identifiable tribes in Minnesota were the Dakota (Sioux) circa 1000 CE and the Anishinaabe (Chippewa, Ojibwe) who arrived in the mid-1700s [22]. Today, Minnesota is home to four Dakota and seven Anishibaabe reservations [23], most of these communities being located in rural or frontier Minnesota counties. In contrast to these early inhabitants whose ancestors have lived in Minnesota for hundreds of years, today foreign immigrants are arriving in Minnesota in increasing numbers [24]. Many of these new arrivals settle in communities outside of the Twin Cities Metro Area [25]. This spectrum of diversity underlies the importance for healthcare providers to learn to provide culturally-responsive care [26]; therefore, students in the course learn about Indigenous people or foreign-born immigrants they might encounter if practicing in rural Minnesota. Each student is assigned a particular culture (not their own), and through readings about and/or interviews with members from that culture prepares a brief presentation they share with the class during a live videoconference session. Again, because this is a HyFlex course a student who knows in advance that they will be unable to attend class when they are scheduled to present are able to pre-record their presentation. Pre-recorded student presentations are played during the live course session. This exercise constitutes 16.7% of the course grade.

As students in this course are training to become pharmacists, they interview pharmacists who currently practice in rural communities (or who have practiced in a rural community in the recent past). These interviews supplement the rural pharmacy practice stories provided by the instructor, the Resident, and the pharmacists who present during class videoconference sessions. Most, but not all, of the pharmacists who participate in the course are the instructor’s former students from the UMN College of Pharmacy, Duluth. In addition to pharmacists with practice experience in rural Minnesota, pharmacists in the instructor’s circle of contacts from rural Alaska, Wisconsin and Michigan have participated in the course, as have pharmacists from four different rural Indian Health Service [27] /Tribal Health Clinics. Potential pharmacist participants are contacted by the course instructor before the course begins to gauge their interest and willingness to participate in a live class session or be interviewed by the students, and are provided with the list of interview questions that will be asked. Characteristics and practice settings of the pharmacists who participated during the first two offerings of the course are presented in Table 1.

**Table 1:** Characteristics and Practice Settings of Rural Pharmacists Participating in a Rural Pharmacy Elective

Practice setting	Number of pharmacists	Female/ Male
Community Pharmacy (Independent or regional chain)	12	9/3
Critical Access Hospital (Ref. [28])	4	3/1
Health System Hospital	12	7/5
Indian Health Service (Ref. [26, 27])/Tribal Health Clinic	9	7/2
Other (Student Health Clinic, Internet Health)	2	0/2

The instructor assigns the students to interview teams of two to three students who conduct structured interviews with the rural pharmacists who practice in community, critical access hospital [28], health system hospital or Indian Health Service/Tribal Health settings. Each student is assigned to one team to interview a community pharmacist, and then to a different team to interview the health system pharmacist. Where possible, teams are structured to reflect gender diversity and include students from different years in the pharmacy program. Each student team contacts their assigned pharmacist and schedules a telephone or videoconference interview. Interviews are intended to last no more than 30 min, but oftentimes go longer as the conversations between the students and the pharmacist range far beyond the structured questions provided by the instructor.

Each student submits written summaries of their two interviews. Each interview team provides informal presentations about their interviews to the class during a live videoconference class session. Each interview assignment constitutes 16.7% of the course grade.

At the end of the course, students once again complete the 7-point Likert-type questionnaire regarding their interest in possibly practicing in a rural community. The numerical results from this questionnaire are compared to the numerical results of the interest questionnaire that the student completed at the start of the course. Each student also writes a brief reflection paper regarding what they learned in the course about practicing pharmacy in a rural community, and what aspect of the course they found most interesting/helpful in their learning. As with the similar assignments at the beginning of the course, the final questionnaire and final reflection paper constitutes 8.37% of the course grade.

### Rural pharmacy elective-topics

Topics presented in the course are listed in Table 2. Topics for didactic sessions early in the course are based on selected chapters from the textbook required for the course, *Foundations of Rural Public Health in America* (2022), by Joseph N. Inungu and Mark J. Minelli [20]. The course also features interdisciplinary and interprofessional components. As noted earlier, one guest presenter is a PhD Biologist employed by one of Minnesota’s American Indian tribes. That individual addresses Climate Justice, explaining the impact of climate change on rural Indigenous communities. Also as noted earlier, a group of rural Advanced Practice Nurses in different subspecialties present a panel session addressing the challenges faced by the communities they serve, and describe how they interact with rural pharmacists in their communities.

**Table 2:** Topics in a Rural Pharmacy Elective

Definitions of Rural
Rural Lifestyles and Health Disparities
Cultural Influences on Health
History of Rural Health & Rural Health Transitions
Guest Presentation: Great Practices in the Great Land (Alaska)
Guest Presentation: Pharmacy Practice in Isolated Tribal Communities (U.S. Indian Health Service/Tribal Health Clinics)
Guest Presentation: Interprofessional Teams in Rural Health
Guest Presentation: Climate Justice and Indigenous Communities
Guest Presentation: Critical Access Hospitals
Student Presentations: Indigenous and Immigrant Residents of Rural Communities
Guest Presentation: Pharmacy Practice in Rural Health Systems
Guest Presentation: Community Pharmacy Practice in Rural Communities
Instructor’s and Teaching Assistant’s Personal Rural Pharmacy Practice Stories

### Assessing course outcomes

The percentages of students enrolled in the course on each campus who reported growing up in a rural community or having spent considerable time visiting relatives who lived in rural communities were compared using Fisher’s exact test [29]. For students completing rural interest questionnaires both at the beginning and the end of the course, rating scores from both years and both campuses were combined and paired. Given the ordinal nature of the data, beginning/end of course ratings were evaluated using the Wilcoxon signed-rank test [30]. A two-tailed alpha value of 0.05 was selected as the criterion to indicate significance in all numerical comparisons.

## Results

For the first offering of the course in Spring, 2022 a total of 25 students completed the course. Spring 2023 had 17 students in the course. The demographics of the students in these two cohorts are summarized in Table 3.

**Table 3:** Demographics of Students Enrolled in Rural Pharmacy Elective Course

	Spring, 2022		Spring, 2023	
Campus	Minneapolis	Duluth	Minneapolis	Duluth
Completed Course	13	12	12	5
Gender: Female/Male	5/8	6/6	9/3	4/1
Rural upbringing^a^	5	6	5	3

^a^Either born and raised in a rural community or experienced extended visits with relatives who lived in a rural community

Between the first two offerings of this course, 25 students on the Minneapolis campus enrolled in the course. Of these 25, 10 (40%) reported growing up in a rural community or having spent considerable time visiting relatives who lived in rural communities. Among the 17 Duluth students enrolled in the course between the two years, nine (53%) reported having grown up or otherwise spent considerable time in rural areas. This difference was not statistically significant.

**Fig. 1:**
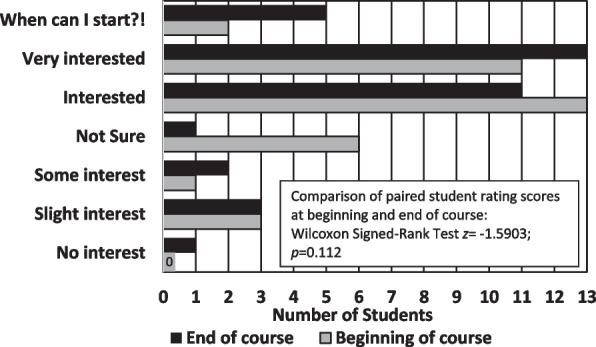
Interest in Practicing Pharmacy in Rural Communities (*n* = 36)

At the beginning and end of the course, students rated their interest in living/practicing in rural community using a 7-point Likert-type scale ranging from “1-No interest” to “7-When can I start?!” The results from the 36 students who completed both the pre and post questionnaires are presented in Fig. 1.

The total net change in Rural Interest scores across all students completing both questionnaires was + 5 in 2022 and + 2 in 2023, some student scores increasing, others decreasing, and still others remaining the same. Results of the Wilcoxon Signed-Ranks Test were non-significant (*z* = -1.5903; *p* = 0.112).

The largest change in scores occurred in the “Not sure” category (middle choice), with only one student remaining unsure of their interest in practicing in a rural community at the end of the course compared to six students at the beginning of the course. Four students who selected “Not sure” at the start of the course expressed lower interest in practicing in a rural community at the end of the course, one of these students moving down three levels from “Not sure” to “No interest.” One student who had selected “Interested” at the beginning of the course also dropped three levels at the end of the course to “Slight interest.” In contrast, several students who had selected “Interested” at the start of the course moved up to “Very Interested” or “When can I start?!”.

**Fig. 2:**
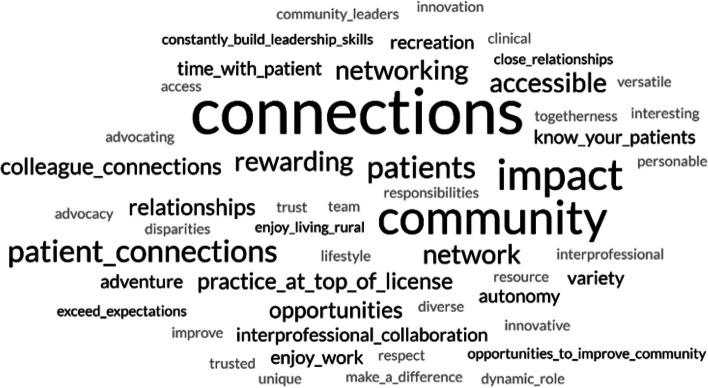
Word cloud featuring perceived benefits of living and practicing pharmacy mentioned in Rural Pharmacy students’ end-of-course reflection papers. “Courtesy of FreeWordCloudGenerator.com”

**Fig. 3:**
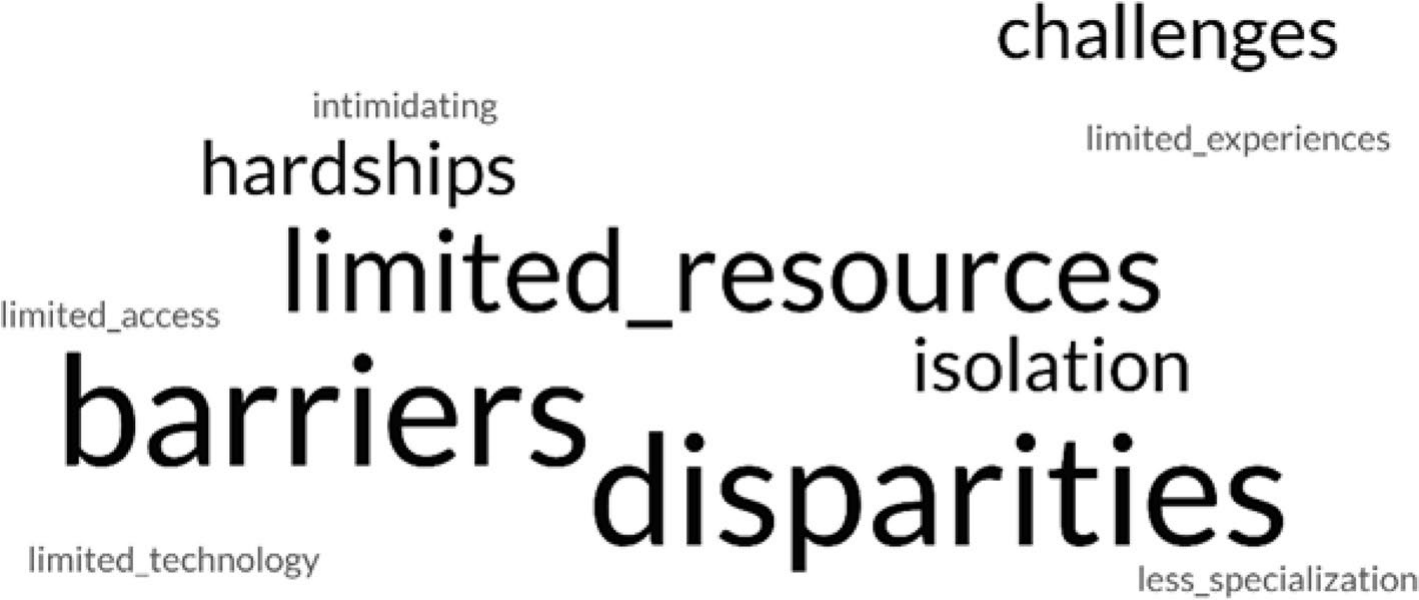
Word cloud featuring perceived challenges of living and practicing pharmacy mentioned in Rural Pharmacy students’ end-of-course reflection papers. “Courtesy of FreeWordCloudGenerator.com”

At the end of the course, students were asked to reflect on the impact of the course on their interest in practicing pharmacy in a rural community. Among the 42 students enrolled in the course during the first two years, 25 students in their reflection papers explicitly expressed appreciation for being able to interview pharmacists currently practicing in rural communities, while 20 explicitly expressed appreciation for having rural pharmacists and other professionals as guest speakers during class sessions. Two word clouds were generated from students’ reflection papers, one based on student perceptions of the benefits of living/practicing in a rural community (Fig. 2), and the other based on student perceptions of the challenges of living/practicing in a rural community (Fig. 3).

Representative student comments excerpted from their reflection papers regarding what they had heard from rural pharmacists who participated in the course are provided below. Each student’s comment is followed by that student’s final rating of their interest in practicing pharmacy in a rural community (1 = No interest, 7 = When can I start?!):

*Before this course I had no interest in practicing rural before but now I’d at least entertain the idea after speaking and interviewing pharmacists that did or currently practice there.* (Student selected ratings of 1 and 2)

*Hearing so many amazing stories, pharmacists are truly more than just “pill counting” because a single pharmacy can connect them with other rural health professionals, expanding the capabilities of rural pharmacists….* (2)

*If you can dream it you can do it in rural pharmacy.* (5)

*It was great to have [the pharmacist I interviewed] in my network, as [they] said I can contact [them] anytime with questions outside… [of] my interview. I learned that having many contacts in your network, especially in rural areas, is so important….*(6)

*This class stimulated a future career interest that I already had, but was not sure exactly how to get started and who to ask if I had any questions. I feel like I now have many resources to reach out to when it comes to my future career, which makes me incredibly happy and comfortable.* (7)

Students also expressed appreciation for other aspects of the course, whether the students were interested in practicing in a rural community at the end of the course or not. Again, each student’s comment is followed by that student’s final rating of their interest in practicing pharmacy in a rural community (1 = No interest, 7 = When can I start?!):

*Even if I do not practice as a rural pharmacist, I will value the exposure and learning that has come from the topics covered in this course.* (3)

*To be frank, I never even entertained the idea of practicing as a rural pharmacist. I’ve always wanted to work in an urban ambulatory care setting…. I did not expect the class to be as eye opening as it truly was…. I’m much more open to serving in a rural community and may consider it strongly*. (3)

*It would be a huge adjustment to move to a rural area since I have grown up in [an urban community] my whole life. I want to work in a rural community since it is rewarding, but it is difficult to leave family behind and essentially start a new life with new people.* (4)

*This is a rural pharmacy class, but it did not feel biased towards only working rural…. I came into this class knowing that I had an interest in rural pharmacy, but I did not expect to come out of this class even more interested in what rural areas have to offer.* (6)

*Before starting this course, I knew that I wanted to practice pharmacy in a rural community…. Many times during this course we stated, “When you’ve seen one rural community, you’ve seen one rural community.” I did not know how true this statement was before this course…. Despite their vast differences, one common underlying theme is the health disparities seen in rural areas.* (7)

## Discussion

It is important that health professions students be introduced to rural culture, even if they are “never” going to live/practice in a rural community themselves. With 5–64% of states’ populations living in rural communities [1], the odds are good that at some point in their careers, health professionals living in large urban centers are going to care for patients who have come from rural communities to receive more specialized care than is available locally [31]. Being introduced to rural culture can help students provide more culturally responsive care [32] to patients from rural communities during their careers.

The purpose of this course was to introduce pharmacy students to the advantages and challenges of practicing and living in rural communities. The course was not intended to “change hearts and minds” of students regarding their possible interest in practicing in a rural setting, and as can be seen from the results, students’ “interest in rural” ratings collectively neither significantly increased nor decreased between the beginning and the end of the course. Regardless, from comments in their reflection papers students generally appreciated the course, finding the interviews with rural pharmacists to be particularly valuable. This finding was heartening to the instructor who was initially concerned about the amount of out-of-class work being asked of the students.

Likewise, guest presenters who participated in the live class sessions and pharmacists interviewed by the students informally expressed their satisfaction with participating in the course, and expressed gratitude that this course was being offered. One pharmacist who previously practiced in a remote Alaska community but had recently moved to a major urban center in the “Lower 48” (Alaskan reference to states in the contiguous United States south of the 49th Parallel) expressed how much they enjoyed sharing their stories with the Rural Pharmacy students. The students with which this pharmacist currently works all desire to practice in large urban centers and are not particularly interested in hearing about the pharmacist’s experiences practicing in small, isolated communities. Another pharmacist noted that they really appreciated joining the students virtually in the live classroom, and was going to recommend this approach to other pharmacy schools with which they work as a way to generate interest in rural pharmacy in general, as well as interest in their particular pharmacy as a clinical rotation site.

A few changes were made in the roster of pharmacists participating in the course from year to year; however, most of the guest speakers and pharmacists who were interviewed by the students participated in the course both years. Another change being considered for the next offering of the course is to add a live videoconference session with a Minnesota Department of Agriculture “Farm Counselor” (a Licensed Professional Counselor) who makes in-person “farm calls” to address farm families’ mental health needs within the unique context of farm culture [33] (MN Dept of Ag, 2023).

## Conclusion

A course specifically addressing the benefits and challenges of practicing pharmacy in rural communities was well-received by pharmacy students enrolled in the course, and by the rural guest presenters and rural pharmacists who were interviewed by the students. Even students who have little interest in living or practicing in a rural community can benefit from being introduced to rural culture, helping all students provide more culturally-responsive care for patients from rural communities.

## Data Availability

The data analyzed during the current study are not publicly available due to stipulations in the U.S. Family Educational Rights and Privacy Act (FERPA), but are available in de-identified form from the corresponding author on reasonable request.

## Abbreviations

CE: Common Era
Km: Kilometers
P1, P2, P3: Students enrolled in years 1, 2 or 3 of the Doctor of Pharmacy (PharmD) program
PGY1: Post-Graduate Year 1
PharmD: Doctor of Pharmacy
PhD: Doctor of Philosophy
PPPS: Pharmacy Practice and Pharmaceutical Sciences
TA: Teaching Assistant
UMN: University of Minnesota
U.S.: United States of America
